# Centring a critical medical anthropology of COVID-19 in global health discourse

**DOI:** 10.1136/bmjgh-2021-006132

**Published:** 2021-06-14

**Authors:** Jennie Gamlin, Jean Segata, Lina Berrio, Sahra Gibbon, Francisco Ortega

**Affiliations:** 1 Institute for Global Health, UCL, London, UK; 2 Departamento de Antropologia, UFRGS, Porto Alegre, Rio Grande do Sul, Brazil; 3 Departamento de Antropologia, CIESAS Unidad Regional Pacífico Sur, Oaxaca, Mexico; 4 Anthropology, UCL, London, UK; 5 ICREA (Catalan Institution for Research and Advanced Studies), Barcelona, Spain; 6 Medical Anthropology Research Centre (MARC), Universitat Rovira i Virgili, Tarragona, Spain

**Keywords:** COVID-19, health policy, public health

**Summary box**

- From its transnational positionality global health homogenises the COVID-19 pandemic as a predominantly biomedical and public health problem, onto which the social sciences are frequently outside looking in.
- We argue for the inclusion of critical medical anthropology in global health explanatory models of COVID-19, side by side and in equal measure, with important biomedical and public health responses.
- The theory and methods of critical medical anthropology, particularly those from the Global South, centring on the political economy of health will keep the structural determinants of health and social justice at the centre of global health ontologies of COVID-19.
- The methods and theory of anthropology would bring an understanding of how the Anthropocene epoch that links environmental, animal and human health has contributed to the emergence and spread of COVID-19.
- Critical medical anthropology emphasises how the neoliberal economic system continues to pattern the pandemic though Trade Related Aspects of Intellectural Property Rights (TRIPS) regulation of vaccines and the unequal distribution of mortality within and between nations—among other factors.
- Southern experiences of the pandemic are less responsive to biomedical solutions.
- We draw on experiences of COVID-19 in Brazil and Mexico, with weaker health systems and greater burdens of non-communicable diseases, to evidence how southern pandemic experiences, where higher than usual mortality from other causes is a major contributor to excess deaths during the pandemic, require different illness explanatory models and responses.
- Repetitions of historical experiences of ethnocide with mortality rates up to 50% higher among indigenous underline the importance of decolonisation in global health.
- Politics is a primary structural determinant of health and we argue for the recognition of this within global health policy and governance to bring political accountability to the discussion table.

## Introduction

The disciplines of biomedicine and global health have been at the epicentre of understanding and finding solutions to the current COVID-19 pandemic. We are thankful for the record-breaking speed of vaccine development, the meticulousness with which the virus is being tracked in order to identify and respond to new variants, developments in hospital care practices and treatments that have contributed to bringing down the case fatality rate and to the breadth of research analysing sex and gender differentials, reasons for the over-representation of black and ethnic minority groups and wider social determinants of COVID-19 mortality. However, global health from its transnational positionality almost always reproduces, in local situations, a ‘global’ coronavirus-centred framework that homogenises the pandemic from a predominantly biomedical perspective, of which the social sciences are frequently outside looking in.

Hetan Shah, Chief Executive of the British Academy, recently made the important case for listening to Social Sciences, Humanities and the Arts for People and the Economy to understand human behaviour, motivations and culture and their role in the pandemic.^1^ As social scientists we applaud this call, yet as medical anthropologists, variously informed by social medicine and epistemologies of the south, we see the dangers of a narrow cultural or behaviourist focus, including analyses that divide nature and culture or environmental, animal and human health. We are also extremely concerned about the coloniality—which defines colonialism as an ongoing process as opposed to an event in the past—of the production and distribution of knowledge about and relating to COVID-19, and the marginalisation of illness experiences from the Global South in the generation and promotion of COVID-19 ontologies, explanatory models and responses. Perhaps most striking however is how, despite a growing public recognition of the way that health inequalities are enmeshed with and have been deepened by the pandemic, there is an almost complete absence of meaningful and impactful reflection about the structural causes that specifically point to the role of the global political economy in shaping the distribution and rates of mortality. As Cousins *et al* note,^2^ epidemiology’s social determinants of health (SDH) framework has ‘sanitised’ the structural determinants model by overlooking demands for health justice. We argue that the theory and methods of critical medical anthropology (CMA), with its focus on the *political economy of health*, are needed to keep structural determinants and social justice at the centre of global health explanations of this and future pandemics.

## Critical medical anthropology: a political economy of health

The structural determinants of health are the causes of the causes. They are the social, political and economic forces that drive inequalities and are determined by people and institutions who hold power. As opposed to concentrating on specific risk factors related to living and working conditions such as poverty or education, as the SDH emphasises,^3^ structural approaches speak to the idea that systemic factors ‘drive, promote and reinforce inequalities’, through the process of social *determination* of health^4^ (p 1). Such concepts are more closely aligned with southern theories such as Latin American social medicine and collective health,^5^ and the concept and social emancipation practice of *buen vivir* (good living), with its origins in the Quechua word sumak kawsay.^6 7^ These counterhegemonic ontologies that speak to a different set of solutions based on social arrangements, are rarely given prominence alongside biomedical and dominant global health and development frameworks. As one of the leading anthropologists of our times, Anna Tsing argues, the Global is a homogenising category based on Western worldmaking, and not a structure that speaks to cultural diversity.^8^

CMA is a branch of anthropology which considers the political economy of health and social inequality in people’s lives. Centring a CMA of COVID-19 means asking, for example, how universals, such as capitalism as the naturalised social and economic order of globalisation, put human societies at increased risk of zoonoses through habitat destruction. We now know that COVID-19 is likely to be only the most recent of many such pandemics this century. Anthropological research using multispecies or more than human approaches is of central relevance for their focus on the dense entanglements between human and animal health.^9 10^ Nevertheless, such perspectives which identify the ‘capitalocene’, the capitalist world ecology premised on resource exploitation and extraction,^11^ as a prime determinant of disease distribution, remain largely excluded from global health discourses around COVID-19.

Another such example of the role of global political economy is that in spite of being backed by more than 100 developing countries, the World Trade Organization did not agree to waive an intellectual property TRIPS for COVID-19 vaccines. Instead, the global response has followed the philanthrocapitalist COVAX initiative led by Gavi, the WHO and Coalition of Epidemic Preparedness Innovations, which at the time of writing had only managed to procure 1.1 million doses of the vaccine in contrast to the 4.6 billion purchased by high-income nations.^12^

## Brazil and Mexico: southern experiences of a global pandemic

Canada, the USA and UK are leading the way in global vaccine inequality with orders in excess of nine, seven and five doses per person, compared with countries such as Brazil and Mexico with orders of around one dose per person.^13^ Despite Brazil’s robust universal healthcare system and long history of successful immunisation programmes, vaccine hesitancy and politicisation by the Bolsonaro government resulted in roll-out that was initially ‘painfully slow, inconsistent and marred by shortages’.^14^ National and global vaccine policies are legitimated by the globalisation of trade legislation that adheres to the ideology of neoliberalism. This is the coloniality of power at a global scale. Since writing this both Brazil and Mexico have accellerated their vaccine rollout, yet it continues to be the case that vaccination rates in the global north far outweigh those in the global south.

CMA also draws out epistemic hegemony in the treatment of experiences of COVID-19 and the impact of these absences on ontologies of causality and response—these are forms of epistemic violence.^7^ In the UK and throughout most of the Global North, COVID-19 mortality and vaccine hesitancy have been consistently higher among black and minority ethnic groups,^15^ an early finding that has rightly led to considerable research as well as discussions of institutional racism. However, such inequities have taken an entirely other dimensions in Mexico and Brazil, which have also produced two of the highest national death tolls in the world. On 6 April, Brazil recorded an astounding 4211 COVID-19 deaths^16^ in the previous 24 hours, while by 15 March Mexico had recorded 444 722 deaths based on excess mortality, a figure that includes non-COVID-19 fatalities. The cumulative excess death rate in Mexico is 49.9%,^17^ while globally the average is 17%. If Mexico had had this overall average excess mortality, the number of COVID-19 deaths would have been 189 465 fewer.^18^

The Brazilian government’s handling of the pandemic should be understood as an intensification of Bolsonaro’s abdication of responsibility for public health governance, itself defined by consistent scientific denialism, promotion of discredited treatments (hydroxychloroquine), dissemination of fake news and freezing of public health funding. Moreover, while this neglect has far-reaching implications, its most destructive effects are predominantly being felt among black and indigenous communities.^19^ In a repeat of colonial history, alarming death rates among Yanomami leave the Amazon tribe threatened with extinction.^20^ How these stories are articulated in global health discourse defines cause and response, and deaths among Brazil’s black and indigenous populations cannot be subsumed under the general inevitability of excess mortality in marginalised groups. From a CMA positionality, governments’ ability to decide who lives and who dies is necropolitics,^21^ and we argue for a more central implication of political responsibility for deaths in global health framings of COVID-19 causality.

For Mexico, like Brazil, the pandemic has predominantly affected populations who are structurally vulnerable. While at a national level the case fatality rate stands at 9%, among indigenous people this figure is 15%.^22^ Geographical inequalities and the already precarious health infrastructures in rural areas have led to differential regional patterning in the effects of COVID-19.^18^ Yet, indicators used globally to measure impact, such as active cases, mortality, case fatality and hospital occupancy, do not capture the effects of the virus in regions where these data and services are lacking.

The country’s already high rates of chronic diseases have translated into a severe shortage of medical staff, ensuring that the Mexican COVID-19 epidemic has become a generalised health crisis across the full range of illnesses from non-communicable diseases to infections, maternal health and geriatric care. In parallel, and in contrast to a pattern that has not been associated with the European pandemic, by July 2020, COVID-19 had become the principal cause of maternal mortality accounting for 21% of deaths, leading to an increase in the maternal mortality ratio from 33.8 in 2019 to 46.6 by December 2020.^23^ In the absence of clinical services and resources, populations have resorted to varying strategies of self-care to treat COVID-19 as well as ongoing chronic and degenerative conditions. As yet, unpublished data on important qualitative indicators, such as loss of employment and crop production, alarming levels of debt and the collapse of entire economies of tourism that have led to acute impoverishment, will further extend the excess mortality brought by this pandemic (Research in process: ‘Documentation of the effects of COVID-19 in afroamerican and indigenous communities of the Costa Chica of Guerrero and Oaxaca. University of California, Santa Barbara and CIESAS, Mexico with funding from Kellogg Foundation’). These experiences are barely considered in the global panorama and provide further evidence of the fact that regions that initially seemed to have had few deaths are in fact dealing with a multidimensional pandemic with case fatality rates far higher than in metropolitan centres or Western nations and yet to be estimated numbers of non-COVID-19 avoidable deaths. In contrast, the case fatality rate in the UK is currently around 1%.^24^ The wide social determination of COVID-19 that is more apparent in the Global South means the biomedical explanatory models—or aetiologies—and hospital treatment are of less relevance, and other ontologies must be given prominence.

For Shamasunder and collaborators,^25^ the pandemic has exposed the emptiness of the rhetoric of equity in global health. The land border between the USA and Mexico is closed and Mexico operates a vaccination policy based on age and need. Yet paradoxically, wealthy Mexicans can cross the border by air to pay for a vaccine in the USA. Hence, the globally agreed criteria for deciding who is to be vaccinated first are subordinated to economic criteria.^26^ Inequality is the driving force in the pandemic and confronting it requires global cooperation, solidarity, coordination and community participation. A social medicine approach that promotes a more complex understanding of the social can, as Adams and colleagues point out, ‘open up the black box of inequity’,^27^ elucidating the structural determinants and social determination of inequalities and helping to reconceptualise global health.

These examples demonstrate how despite being a catastrophe on a global scale, the pandemic is not a universal phenomenon, nor is it homogeneous. Each outbreak that constitutes it has unique and contingent forms, intensities and qualities, which impel qualitative research efforts.^28^ CMA can give prominence to localised experiences, including the intersections between gender, race and labour; cultural and religious differences, social injustices and environmental inequalities. In so doing, it alters the perception of risk by bringing into view how, for example, conditions of extreme racialised violence, economic insecurity associated with the global narcotics market such as those experienced in Colombia^29^ and Mexico, alter perceptions of the severity of COVID-19.

These are only two examples of nations where the state has taken poor leadership in the response to the pandemic, political situations that have combined with economic weaknesses such as high reliance on the informal sector, pointing to a political economy of COVID-19 causality and response that is heavily determined by neoliberal state structures. As one of the fathers of CMA, Rudolf Virchow (1821–1902) famously declared, ‘Medicine is a social science and politics is nothing but medicine writ large.’^30^ The political economy must be considered as causal and it is no surprise that the rise of populism, which has nurtured COVID-19 conspiracy theories, is also reflected in patterns of high mortality in Mexico, Brazil and the USA.^31 32^

## Centring a CMA of COVID-19 in global health discourse

A political economy approach to COVID-19 would address how historical, unequal and neoliberal arrangements, colonially defined racisms, informal economies and high burdens of chronic diseases intersect as power differentials within the provision of healthcare, enabling a more comprehensive assessment of the impact of the pandemic. Yet these non-Western experiences of COVID-19, alongside explanations that point to the social *determination* of COVID-19, have had little influence on the discourses of global health, which fails to articulate how our global political and economic system is responsible for the magnitude and, to some extent, also the emergence of this pandemic.

By arguing for the centring of CMA of COVID-19 in global health discourse, we do not mean to displace the important biomedical and public health responses we mentioned in the opening paragraph, but to ask that anthropologies of cause and treatment are considered side by side and in equal measure. Advanced biosecurity technologies, which use data mining systems or DNA mapping to track virus strains in real time, are essential. They make these molecular worlds of COVID-19 more and more visible. However, the complexity of a pandemic exceeds viruses and their biological mechanisms of contamination and infection.^33^ The broad qualitative research on the social impacts of the pandemic carried out in Brazil by the Rede COVID-19 Humanidades MCTI^34^ has shown that this overexposure of the pathogen ends up obliterating our critical view on the most ordinary situations of everyday life, which is where and how COVID-19 contamination happens.

## Conclusions

Despite being one of the world’s most important sites of vaccine production, a ‘crime against humanity’ is playing out in India,^35^ the latest epicentre of COVID-19 deaths, where ‘oxygen is the new currency’ and mortality is estimated to be up to 30 times higher than the official count. If mass vaccination is to serve as the short-term solution to this pandemic, its unequal distribution must be addressed immediately. A long-term view cannot continue to ignore the political, structural and colonial determinants. Global health could learn from this wisdom, but to do so it must be prepared to speak truth to power, to reject the taken for granted and articulate the objective and subjective dimensions of life in society.^36^ Politics is a primary structural determinant of COVID-19 mortality and we argue for the recognition of this within global health policy and governance to bring political accountability to the discussion table.

As Singer and Rylko-Bauer suggest,^37^ we need to shift the traditional view of risk groups and behaviours to address risk environments and agents. Such a movement will help us understand the dynamics of the entanglements between materialities, speeches, practices and meanings that highlight the pandemic’s multiplicity and inequality. If the pandemic is not homogeneous, our responses to it cannot be either.

Cousins *et al* 2 argue for a ‘reconceptualisation’ of the architecture of global health as ‘categories, crisis and scaffolding of the Global Health enterprise are transformed’, and to do this we must do away with disciplinary silos and the top-down colonial legacies that privilege the Global North and its knowledge production.^38 39^ Fundamentally confronting the structural inequalities means anthropology and views from the South must not remain critical analysis looking in, but become essential components of this project.

## Data Availability

There are no data in this work.
